# Management of hemodynamically stable wide QRS complex tachycardia in patients with implantable cardioverter defibrillators

**DOI:** 10.3389/fcvm.2022.1011619

**Published:** 2023-01-04

**Authors:** François D. Regoli, Mattia Cattaneo, Florenc Kola, Albana Thartori, Hekuran Bytyci, Luca Saccarello, Marco Amoruso, Marcello Di Valentino, Andrea Menafoglio

**Affiliations:** ^1^Cardiology Service, Ospedale San Giovanni, Cardiocentro Institute, Ente Ospedaliero Cantonale, Bellinzona, Switzerland; ^2^Faculty of Biomedical Sciences, Università della Svizzera Italiana (USI), Lugano, Switzerland; ^3^Department of Internal Medicine, Ospedale San Giovanni, Ente Ospedaliero Cantonale, Bellinzona, Switzerland

**Keywords:** wide QRS complex tachycardia, ICD programming, anti-tachycardia pacing, ICD therapies, treatment of ventricular tachycardia

## Abstract

Management of hemodynamically stable, incessant wide QRS complex tachycardia (WCT) in patients who already have an implantable cardioverter defibrillator (ICD) is challenging. First-line treatment is performed by medical staff who have no knowledge on programmed ICD therapy settings and there is always some concern for unexpected ICD shock. In these patients, a structured approach is necessary from presentation to therapy. The present review provides a systematic approach in four distinct phases to guide any physician involved in the management of these patients: PHASE I: assessment of hemodynamic status and use of the magnet to temporarily suspend ICD therapies, especially shocks; identification of possible arrhythmia triggers; risk stratification in case of electrical storm (ES). PHASE II: The preparation phase includes reversal of potential arrhythmia “triggers”, mild patient sedation, and patient monitoring for therapy delivery. Based on resource availability and competences, the most adequate therapeutic approach is chosen. This choice depends on whether a device specialist is readily available or not. In the case of ES in a “high-risk” patient an accelerated patient management protocol is advocated, which considers urgent ventricular tachycardia transcatheter ablation with or without mechanical cardiocirculatory support. PHASE III: Therapeutic phase is based on the use of intravenous anti-arrhythmic drugs mostly indicated in this clinical context are presented. Device interrogation is very important in this phase when sustained monomorphic VT diagnosis is confirmed, then ICD ATP algorithms, based on underlying VT cycle length, are proposed. In high-risk patients with intractable ES, intensive patient management considers MCS and transcatheter ablation. PHASE IV: The patient is hospitalized for further diagnostics and management aimed at preventing arrhythmia recurrences.

## 1. Introduction

Based on data from a survey performed in the UK (1), the prevalence of ventricular arrhythmias is between 0.25 and 0.5% and higher in patients older than 65 years of age. A minority of cases with wide QRS complex tachycardia (WCT), do not have ventricular tachycardia (VT), but rather a supraventricular tachycardia (SVT). Of these patients only a minority also have an implantable cardioverter defibrillator (ICD). Patients that do have an ICD, are by definition, at higher arrhythmic risk, because they have an underlying arrhythmic heart disease. Cardiac arrhythmic diseases implicate, most frequently the presence of underlying structural heart disease (SHD) or, less frequently, an inherited primary arrhythmia syndromes (IPAS), namely long QT syndrome (LQTS), short QT syndrome (SQTS), Brugada syndrome (BrS), catecholaminergic polymorphic ventricular tachycardia (CPVT), early repolarization syndrome (ERS), or idiopathic ventricular fibrillation (IVF).

In a single-center experience, cumulative incidence of any form of appropriate ICD therapy at 10 years for secondary prevention indication patients was 65% (2). In an another single-center contribution, the rate of ventricular arrhythmic episodes that occurred at 2 years follow-up was roughly 20% in patients with primary prevention indication compared to 37% in patients with an ICD in secondary prevention (3).

As may be extrapolated from the epidemiological data, the prevalence of an incessant, hemodynamically stable WCT in ICD patients is an uncommon event (roughly 0.5–1.0%/year). Preparing the staff through the implementation of some fairly simple measures could be of great assistance for adequate and regular management. The fact that the patient who presents has an ICD, and despite this, presents with sustained WCT, may be a source of concern that the ICD may deliver shocks unexpectedly.

General management of WCT in the emergency setting has been addressed in the most recent European practical guidelines on the management of supraventricular tachycardia (4). WCT may be supraventricular or sustained monomorphic ventricular tachycardia (VT). Although hemodynamically stable VT may not present immediately with signs and symptoms of organ hypoperfusion, the clinical situation may deteriorate suddenly and rapidly, especially in “high-risk” patients with electrical storm (ES). ES is defined as the occurrence of 3 or more distinct episodes of VT within 24 h (5) or incessant VT for more than 12 h (6). In ICD patients who present with a sustained WCT, patient management differs in three ways:

(1) The ICD may deliver unnecessary or inappropriate shock and therefore a magnet may be utilized temporarily to withhold anti-tachycardia therapies.
(2) If no shocks have been delivered, 1 or more of these conditions may be present.
  (a) The arrhythmia cycle length (CL) is below that of the therapy window.
  (b) The WCT is supraventricular and discriminating algorithms have adequately detected and interpreted the arrhythmia.
  (c) Only anti-tachycardia pacing therapies (ATPs) without shocks were programmed in the slower VT window and the device is now in “ATP time out” mode, i.e., a programmable maximum time duration during which ATP was permitted to continue.
(3) Manual programming of ATP bursts, scans and/or ramps to terminate WCT may be programmed, without the need for deep sedation and electrical cardioversion.

This review discusses the diagnosis and treatment by proposing a structured approach for the optimal management of hemodynamically stable sustained WCT in ICD patients. The document is structured in four parts. In the first part, the most relevant measures needed upon presentation of the patient are discussed, namely measures to avoid the delivery of unnecessary/inappropriate shocks, careful interpretation of the ECG, identification of eventual triggers, and patient risk stratification when presentation is ES. Management of patients who present with ES and a “high risk” profile should follow an accelerated management algorithm consisting in pre-alerting a tertiary center. In the second part, preparatory measures are presented, including contacting the anesthesiologist and, if available, the ICD specialist. In the third part, after evaluation, and especially based on whether an ICD specialist is available, the chosen therapeutic approach is applied. In the last part, further diagnostic and therapeutic measures that are usually indicated during the hospitalization of these patients are discussed.

## 2. PHASE I: Presentation of the ICD patient with hemodynamically stable WCT

### 2.1. Early assessment and inhibition of ICD therapies

Upon arrival in the emergency room (ER) or in the intensive care unit (ICU), the patient is immediately placed under continuous monitoring of heart rate, rhythm, and blood pressure. The patient’s hemodynamic status is then assessed. Hemodynamically stability is established in the absence of life-threatening features; specifically, hemodynamic shock (systolic blood pressure < 80 mmHg with signs of peripheral hypoperfusion), syncope, severe heart failure (pulmonary edema or increased jugular venous pressure) and myocardial ischemia (chest pain in patient with known coronary artery disease) are excluded (7). In case one of these conditions are present, measures for rapid conversion to sinus rhythm are indicated consisting in synchronized electrical external cardioversion (5, 7). In patients with ICDs, some caution should be taken in the positioning of external defibrillation patches or when applying external defibrillation chest paddles as discussed below.

**FIGURE 1:**
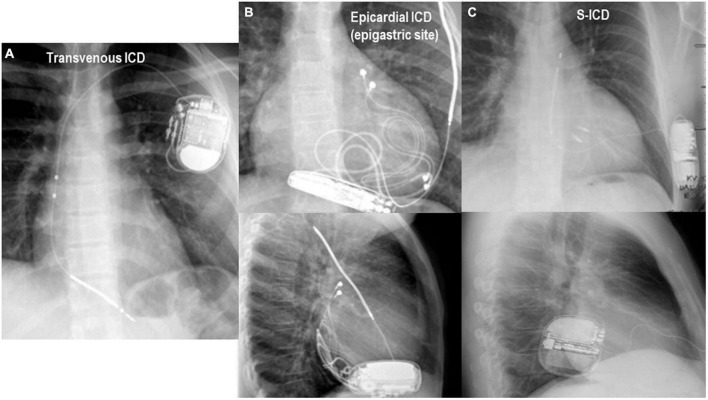
Chest X-ray in anterior-posterior and lateral projections are important for the identification of the type of implantable cardioverter defibrillator (ICD) and for the application of the magnet which should be fixed on top of the ICD can for disabling therapies. **(A)** Panel shows the typical position of a transvenous ICD immediately below the left clavicula. **(B)** Panel shows the radiologic antero-posterior and lateral projections in an 8 year-old girl with an epicardial ICD and the can located in the epigastric area. In panel **(C)**, the typical position of the S-ICD system is appreciated.

After confirming that the patient is hemodynamically stable, an external magnet may be applied on top of the ICD pulse generator to avoid inappropriate/unnecessary therapies. This is especially important in patients who have already experienced 1 or more ICD shocks upon presentation. While the ICD generator can is usually positioned in left subclavicular sub-cutaneous or sub-pectoral positions, the sub-cutaneous ICD (S-ICD) is located along the left midaxillary line. The S-ICD is an extra-vascular “shock box” most often indicated in young adult patients with either IPAS or a form of dilative cardiomyopathy in the absence of a pacing indication for bradycardia (8, 9). This type of ICD has shown reduction of long-term device-related complications (9–11) in different IPAS patient series. In pediatric patients with ICDs, the ICD generator is often located in the upper abdominal quadrants. Figure 1 illustrates different ICD types, namely a transvenous single-chamber ICD, epicardial single chamber ICD, S-ICD and the anatomic position of the ICD can for each type. Magnet application deactivates tachycardia detection and/or anti-tachycardia therapy without influencing bradycardia pacing. Although in most ICDs and CRT-Ds, the pacing mode, sensor function, pacing polarity and intervals remain unchanged, some ICD devices behave differently. Most importantly, in Microport (former Sorin) ICDs, pacing mode, sensor function, pacing polarity and intervals do change during magnet response. Pacing output is increased to 6V @ 1 ms for each chamber, the sensor (R-function) is disabled. If the device is in “Mode switch” pacing is performed according to permanently programmed mode independently of underlying rhythm, and, in CRT-D devices, AV delay does not change, but VV delay is set to 0 ms. Several contributions have extensively described magnet response of ICDs [(12–14); refer to the “Appendix” for further details].

### 2.2. Twelve-lead ECG interpretation algorithms

**FIGURE 2:**
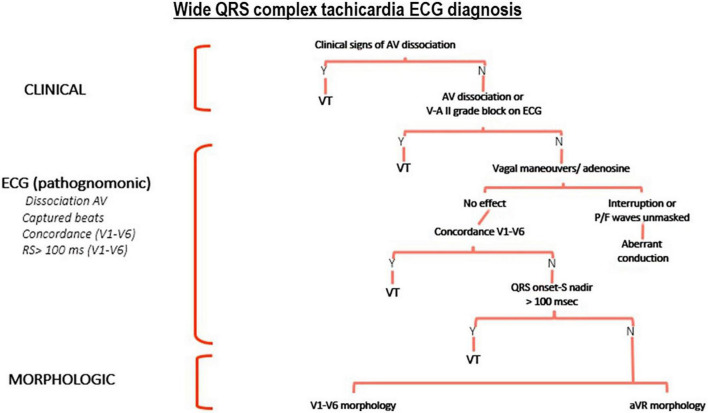
Proposed systematic and structured diagnostic tree for the ECG diagnosis of wide QRS complex tachycardia (WCT). AV, atrio-ventricular; V-A II grade block: retrograde “P” wave is present after the end of the QRS complex with a 2:1 sequence; VT, ventricular tachycardia.

**FIGURE 3:**
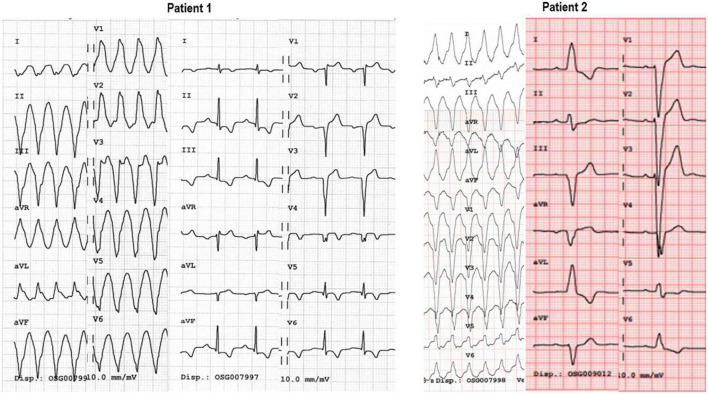
In this 49 year old patient (Patient 1) with a previous antero-septal infarct several years before, WCT at 190 bpm, QRS axis is deviated to the right; atypical RBBB morphology and the presence of a monophasic R wave in aVR indicate VT. The ECG at rest in sinus rhythm, shows the absence of an R wave from V1-V6 indicative of antero-septal transmural necrosis. Patient 2 presents with a WCT at 180 bpm, with a normal QRS axis and typical LBBB pattern in the precordial leads (QS in V1, monophasic R-wave in V6). QRS morphology in sinus rhythm is the same as the one during tachycardia. Intravenous adenosine, allowed to diagnose atrial tachycardia conducting with LBBB.

Once the patient is monitored, a 12-lead surface electrocardiogram is performed (Figure 2). Different ECG diagnostic algorithms have been proposed for the diagnosis of WCT. Some of these algorithms are simple, others are more complex. The most comprehensive and accurate algorithm is, in fact, based on a systematic and structured approach (15) which integrates clinical features (patient history and comparison of a previous ECG for example) (16–18), pathognomonic ECG features for the diagnosis of VT (18), and morphological features of VT in the precordial leads (19) and in a VR (20) (Figures 2, 3).

### 2.3. Laboratory tests and echocardiography

**FIGURE 4:**
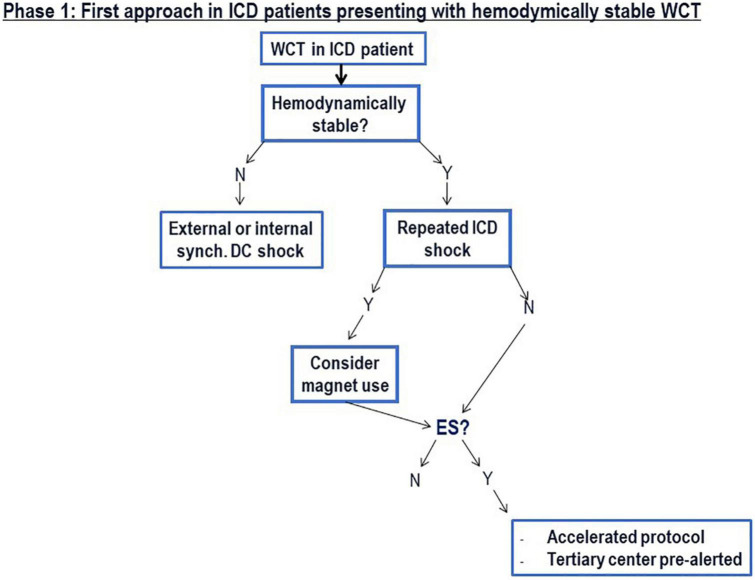
The different steps of the first phase are summarized.

At the same time, a routine blood test is performed including determinations of potassium, sodium, magnesium levels, high-sensitive Troponin, creatinchinase (total and MB), creatinine, glomerular filtration rate, hemoglobin, hematocrit, CRP, D-dimers, BNP, and white blood cell count, thyroid-stimulating hormone. Electrolytic alterations, myocardial injury, renal impairment, anemia, hypovolemia, heart failure, inflammation or infection may all act as triggers, especially in the presence of a vulnerable myocardial substrate (Figure 4).

Depending on the clinical phase, point of care ultrasonography (POCUS), provides further assistance for the identification of potential mechanical triggers (pericardial effusion, signs of acute pulmonary hypertension, etc.) and/or characterization of cardiac chambers, contractility of both ventricles as well as anatomy and function of cardiac valves (21).

### 2.4. Electrical storm: Identification of the “high-risk” patient

It is important to establish whether the current episode of sustained hemodynamically stable WCT is ES, since this situation entails elevated intra-hospital morbidity and mortality, especially in clinically compromised patients. ES is often an arrhythmic situation associated with compromised left ventricular systolic function, with heart failure symptoms, and other comorbidities (22, 23). One study found that in CRT-D patients as well, ES was associated with non-ischemic heart disease, ICD secondary prevention indication, and with persistent heart failure symptoms and low LVEF despite CRT (24). Unfavorable prognosis following ES is determined by low LVEF, ICD secondary prevention indication, advanced NYHA class, and the presence of comorbidities such as chronic obstructive lung disease (25, 26). Therefore, if the patient presents a “high risk” profile, an accelerated management protocol should be activated, that includes pre-alerting a tertiary clinic for eventual urgent therapeutic measures such as transcatheter ablation with or without mechanical cardiocirculatory support (MCS) bridging (see parts “4.4.3 Left ventricle unloading” and “4.4.4 Radiofrequency catheter ablation”).

## 3. PHASE 2: Preparation for therapy

For patient clinical stabilization, several non-anti-arrhythmic measures are taken according to the clinical picture as well as the laboratory results. These include, correction of electrolytes and/or volume depletion, and mild sedation. Sometimes, as is the case with first-aid measures, these may determine interruption of the arrhythmia.

Once the patient is monitored and stabilized, the anesthesiologist/intensive care teams and, depending on available resources, the ICD clinic are notified for the organization of the therapeutic plan. Anti-arrhythmic treatment then follows and mainly depends on the availability of a device specialist for device check and, eventually, delivery of anti-tachycardia ICD-based therapies.

While the same preparatory measures should be followed for the “high-risk” patient with ES, these should be implemented rapidly.

## 4. PHASE 3: Anti-arrhythmic therapies

### 4.1. Conversion to sinus rhythm when device-based therapies cannot be delivered

**FIGURE 5:**
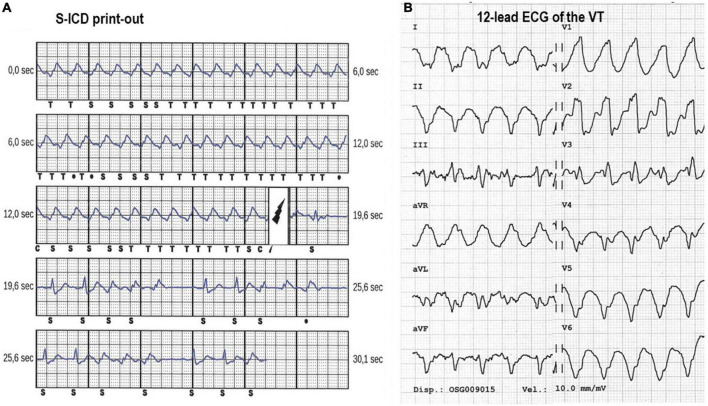
**(A)** Panel shows the print-out of the S-ICD electrogram of a 46 year old patient with dilatative cardiomyopathy. The ventricular tachycardia at 160 bpm with shock therapies set at 200 bpm. The device electrogram (EGM) shows how there is double-counting of the QRS due to T-wave oversensing and a 80 Joules shock is delivered with successful interruption of the arrhythmia. **(B)** Panel shows the ECG of the VT. The patient experienced repeated shocks following gastroenteritis with hypokalemia without loss of consciousness. The arrhythmia was successfully controlled by hydration, intravenous KCl, and intravenous 150 mg of amiodarone (the patient was already under chronic treatment with amiodarone).

Patient treatment depends on the type of ICD and whether a device specialist is readily available. In the event that the device model is an S-ICD, and/or that a device specialist is not readily available, then a stable WCT should be treated according to the proposed management algorithm of the Guidelines (4, 5). In patients with SHD, if there is a low suspicion of reentry SVT or after vagal manoeuvers and adenosine have failed, intravenous amiodarone is the drug of first choice. For the management of hemodynamically stable ES either endovenous amiodarone or non-selective beta-blocker are recommended. In this setting, propranolol is superior to metoprolol (27). Endovenous procainide is also effective for suppression of stable VT, but should not interact with sotalol or amiodarone and is not always available. Less commonly, as second line treatment, the use of endovenous lidocaine (4, 5, 7) (Figure 5) may be considered. Table 1 summarizes the main therapeutical measures indicated in patients with WCT who have underlying SHD.

**TABLE 1:** Therapeutic measures used for conversion to sinus rhythm and for ventricular rate control during hemodynamically stable wide QRS complex tachycardia.

Measures	Dosages	Remarks and precautions
Vagal manoeuvers	/	“Modified” vagal manoeuver is more effective to interrupt AVNRT or AVRT
Adenosine i.v.	6–18 mg	No evidence of pre-excitation at baseline ECG Suspect of AVNRT or orthodromic AVRT
Amiodarone i.v.	300 mg push, followed by 150 mg push 1–2 g/24 h	During ES in SHD
Procainide i.v.	10 mg/kg in 20’	Stable monomorphic VT Caution in pts under amiodarone or sotalol Watch for hypotension and QRS prolongation Limited availability
Propranolol p.os.	40 mg/6 h	ES/VT with stable blood pressure
Metoprolol i.v.	5 mg push every 5’, to max of 15 mg	ES/VT with stable blood pressure
Lidocaine i.v.	1 mg/kg push 1–2 mg/min	2° line therapy in stable monomorphic VT

Listed anti-arrhythmic drugs are those indicated in patients with structural heart disease (SHD). AVNRT, atrio-ventricular node re-entry tachycardia; AVRT, atrio-ventricular re-entry tachycardia; ES, electrical storm; SHD, structural heart disease; VT, ventricular tachycardia.

The management of IPAS deserves specific consideration, because this group includes a wide spectrum of different arrhythmic diseases. For the arrhythmic management of these patients, immediate contact with a specialized tertiary is highly recommended.

In long-QT syndrome (LQTS) stopping QT-prolonging drugs is of pivotal importance. Potassium supplement and spironolactone have been proposed for LQTS2. Class Ia Mexiletine and class Ic Flecainide have been proposed for LQTs3 (enhanced sodium channel function) (28, 29). Available data suggest the efficacy of Quinidine therapy in short-QT syndrome, by prolonging the QTc interval (30–32). Drug efficacy of Quinidine is maintained in the long-term (33). Class Ic Quinidine as well as isoprotenerol or temporary atrial overdrive pacing in patient with dual-chamber ICD may interrupt an incessant or recurring VT in Brugada Syndrome and early repolarization syndrome. These measures prevent premature ventricular beats during bradycardia and reduce early after-depolarization (34). Moreover, aggressive control of temperature is a significant part of the comprehensive management for patients with Brugada syndrome (34–37). Betablockers, sedation as well as flecainide have been implemented for patients with catecholaminergic polymorphic ventricular tachycardia (38–40).

Should the patient require external electrical cardioversion because of onset of hemodynamically instability, because other measures have failed, or because this is the preferred approach, then caution should be taken in the positioning of the defibrillation patches and/or paddles (41). Positioning of the patches should be either in apex-anterior or apex-posterior positions in such a way that the defibrillation current vector is distant from the defibrillator generator. In patients with an S-ICD, the patches should be placed anteriorly along the right parasternal line and dorsally left of the thoracic spine.

High-energy synchronous shock should be delivered. If patches are not effective, suspect inadequate contact or vector and switch to manual cardioversion with the paddles. Conversion to sinus rhythm of WCT through external electrical cardioversion is safe and effective if applied correctly.

### 4.2. Conversion to sinus rhythm by device-based therapies

The availability of a device specialist allows ICD interrogation. The presence of a transvenous ICD during a hemodynamically stable WCT has several implications. First, confirmation of the diagnosis of the WCT may be achieved; second, delivering atrial or ventricular pacing burst or ramps may effectively terminate the arrhythmia; third, effective ATP algorithms may then be programmed to treat future recurring arrhythmic events. Importantly, S-ICD is a “shock-only” ICD device and cannot deliver ATP.

#### 4.2.1 Fundamental concepts and clinical data on non-shock-based ICD treatment of arrhythmias

Whether the origin of the sustained WCT is supraventricular or ventricular, the underlying mechanism is most likely reentry. Being able to terminate a reentry arrhythmia by delivering a train of impulses is based on the concept that effective entrainment, i.e., the train of impulses has entered the excitable gap of the reentry circuit and “unpins” the reentry rotor through entrainment, creates another pacing-induced and sustained rotor, which is interrupted when pacing is stopped. While in theory this principle is quite effective to terminate slower supraventricular and ventricular arrhythmias, its effectiveness is limited for higher frequency VT and for atrial fibrillation (multiple rotors and wave fronts) (42–44).

Clinical data concerning the termination of SVT using ICD-based pacing algorithms is limited to experience and knowledge derived from electrophysiological studies. Reentry atrial tachycardia (AT), atrioventricular node reentry tachycardia (AVNRT), and atrioventricular reentry tachycardia (AVRT) are amenable to effective atrial burst pacing because the arrhythmia cycle length (CL) is not too short and it is easier to penetrate into the circuit for entrainment by using bursts that are 15–20% shorter than the SVT CL.

Importantly, short bursts of ventricular pacing at a programmable shorter cycle length than the arrhythmia may effectively terminate VT (45, 46). Different ATP modalities may be programmed, including fixed rate burst and ramp pacing ATPs. Ramp designates a sequence of programmed pulses in which each pulse is delivered at a progressively slightly shorter interval than the previous one.

Anti-tachycardia pacing in the VT window (<185 bpm) are usually programmed in secondary prevention patients with previous known stable monomorphic VT (47, 48). The first sequences are usually bursts with pulse increments between each scan, followed by several trials of ramps with progressively shortening of CL between pulses. The study by Schaumann et al. (49) tested an empiric ATP sequence scheme in patients implanted with an ICD in whom no stable VT was inducible during an EP study. In this study, most of the patients were implanted in secondary prevention and in more than half of the patients ICD therapies were programmed in the VT window (<185 bpm). The empiric ATP algorithm consisted in three trials of autodecremental ramps with 8–10 pulses, 8 ms decrement between each pulse, starting with CL of 81% of the detected VT. The minimal interval between pulses was set at 200 ms. The effectiveness of this ATP scheme to terminate VT was 90%, while 5% of patients experienced acceleration following ATP delivery.

Concerning ATP delivery for the treatment of fast ventricular tachycardia (FVT > 185 bpm), several large multicenter clinical trials have shown the safety and the effectiveness of programming 1 ATP of eight pulses, at 85–88% of VT CL (50, 51), in primary prevention, including CRT patients with non-ischemic heart disease etiology (52) and in secondary prevention patients (53), showing a significant reduction of any ICD shock. Shock reduction in these studies was not only determined by the effects of a standard ATP algorithm for the treatment of FVT, but this ATP algorithm was programmed in combination with long detection intervals as well as SVT discrimination features.

Later multicenter studies have demonstrated the relative value of ATP for VT and FVT termination, regardless of indication and underlying heart disease (46, 53). Some additional studies have further demonstrated that delivering a single biventricular ATP burst in patients with CRT-D and ischemic etiology (46) may effectively interrupt FVT and reduce shocks.

#### 4.2.2 Anti-tachycardia pacing delivery in the emergency setting

As the device is being interrogated during the arrhythmia, it is important to have identified the type of device (S-ICD, transvenous single-, dual-chamber, or CRT-D device) and to have knowledge of the device electrophysiological (EP) features (Table 2). Apart from single chamber ICD devices, some other manufactures do not offer the possibility to stimulate the atrium. This is the case of MicroPort devices. In patients who present such devices, they should be treated like S-ICD recipients.

**TABLE 2:** Available implantable cardioverter defibrillator (ICD) device features for each manufacturer for stimulation of the atrium and/or the ventricle during tachycardia.

Manufactures	Function on the programmer	Atrial therapy*§	Manual burst method in atrium (A) and ventricle (V)	Commanded bursts/Programmed PES in atrium (A) and ventricle (V)	Biventricular bursts**	Comments
Abbott	NIPS	√	√(A) √(V)	√(A) √(V)	X	
Biotronik	A: NIPS V: DFT (EPS/ATP)	√	√(A) √(V)	√(A) √(V)	√	
Boston Scientific	EP tests	√	√(A) X(V)	√(A) √(V)	√	For termination of atrial arrhythmias high rate pacing (50 Hz) may be delivered
Medtronic	EP study	√	X	√(A) √(V)	√	
MicroPort	X	X	X	X	X	ATPs from the V according to standard procedure under “Tachycardia” heading

A, atrial chamber; V, right ventricular chamber; √, feature is available in recent generation ICD models. X, feature is not available.

*Dual-chamber and CRT-D models.

**Recent generation CRT devices (Medtronic, Biotronik, and Boston Scientific) fixed bursts and programmed electrical stimulation may be performed using biventricular stimulation.

^§^PM-dependent ensure that back-up pacing is activated.

**FIGURE 6:**
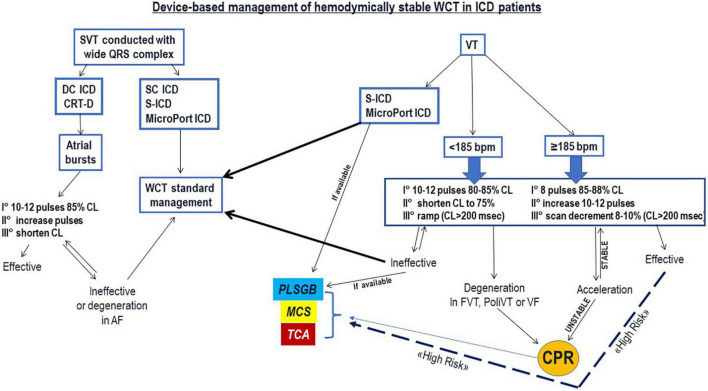
The figure proposes a device-based management algorithm for the treatment of wide QRS complex tachycardia (WCT) in ICD patients, based on the type of ICD, specific ICD features according manufacturer, and stimulation therapies delivery from the atrium (in case of supraventricular tachycardia) or from the ventricle (in case of ventricular tachycardia). AF, atrial fibrillation; CL, cycle length; CPR, cardio-pulmonary resuscitation; CRT-D: cardiac resynchronization ICD; DC ICD, dual-chamber ICD; FVT, fast ventricular tachycardia; MCS, mechanical cardio-circulatory support; PLSGB, percutaneous left stellate ganglion block; PoliVT, polymorphic ventricular tachycardia; S-ICD, subcutaneous ICD; SC ICD, single-chamber ICD; SVT, supraventricular tachycardia; TCA, transcatheter ablation; VT, ventricular tachycardia.

The possibility to deliver rapid burst pulses manually (Abbott and Biotronik ICDs) from both the atrium and the ventricle, allows to rapidly activate ATPs and may avoid excessive stimulation, thus reducing the risk of arrhythmia acceleration or degeneration in AF for the atrium or VF in the ventricle, respectively. Although there are no data to support this, in experienced hands this feature is preferred. Figure 6 proposes a decision tree for the WCT management in ICD patients considering different ICD models, ATP algorithms and VT cycle length. Table 3 proposes a step-wise approach for the programming of ATPs based on VT rate.

**TABLE 3:** Proposed step-wise approach for the programming of anti-tachycardia pacing therapies (ATPs) based on the rate of the ventricular tachycardia.

	VT < 185 bpm	FVT ≥ 185 bpm
Burst	8–10 pulses at 75–85% VT CL	Eight pulses at 85–88% VT CL
Scans	Decrement scans by 5% until 75%	I° increment pulses to 10–12 II° decrement scans by 5% until 78%, and for each scan with CL shortened increase pulses to 10–12, before further shortening*
Ramps	I° 10 pulses, 81% VT CL, 8 msec decrement* II° 10 pulses, 75% VT CL, 8 msec decrement* III° 10 pulses, 75% VT CL, 10-12 msec decrement*	Ramps are not recommended in FVT

*Minimum CL between pulses > 200 msec. CL, arrhythmia cycle length; FVT, fast ventricular tachycardia; VT: ventricular tachycardia.

##### 4.2.2.1 Burst pacing from the atrium to terminate SVT

During reentry supraventricular tachycardia, delivering burst pacing manually or through programmed burst algorithms, by starting with 80–85% CL is recommended. By keeping the same interval, therapy effectiveness may be obtained by lengthening the train of pulses and increasing energy output. If the arrhythmia persists, shorten CL < 80%. In these cases, if the SVT degenerates in AF the ventricular response is usually lower and partial clinical benefit may result, and therefore there is less concern about the effects of acceleration at the level of the atrium.

##### 4.2.2.2 Anti-tachycardia pacing delivery for VT < 185 bpm

Even though the ATP scheme proposed by Schaumann (45) is highly effective, current ICD programming guidelines recommend to begin by delivering 8–10 pulses at 75–85% CL and progressively to lengthen pulses and shorten CL until 75% (43, 44). If after 4–5 trials of ATP the arrhythmia persists unchanged, then delivery of ramps according to the Schaumann scheme (10 pulses, 81% ramp, with 8 ms decrement between pulses) ensues, ensuring that a minimum CL interval between pulses ≥ 200 ms is programmed.

##### 4.2.2.3 Anti-tachycardia pacing delivery for VT ≥ 185 bpm

During sustained FVT (≥185 bpm), consider starting with eight pulses, at 85–88% CL; if ineffective, strengthen by increasing pulse number to 10–12 and, lastly, program scans with 5% decrement, respectively. Programming ramps is not recommended for FVT, and decrementing should be made with caution especially in VT with high rates. In patients with ischemic etiology and CRT-D devices with biventricular burst capability (Medtronic, Biotronik, and Boston Scientific CRT-D devices), consider delivering biventricular bursts following the same sequence of attempts (54).

### 4.3. Anti-tachycardia pacing do not interrupt supraventricular tachycardia

When vagal maneuvers as well as repeated ATPs do not allow termination of WCT, further treatment depends on the persistence of hemodynamic stability, and the severity of underlying heart disease. When aberrant SVT is suspected, and no structural heart disease known, procainamide can be used (4, 5).

### 4.4. Anti-tachycardia pacing do not interrupt ventricular tachycardia

#### 4.4.1 First-line management

**FIGURE 7:**
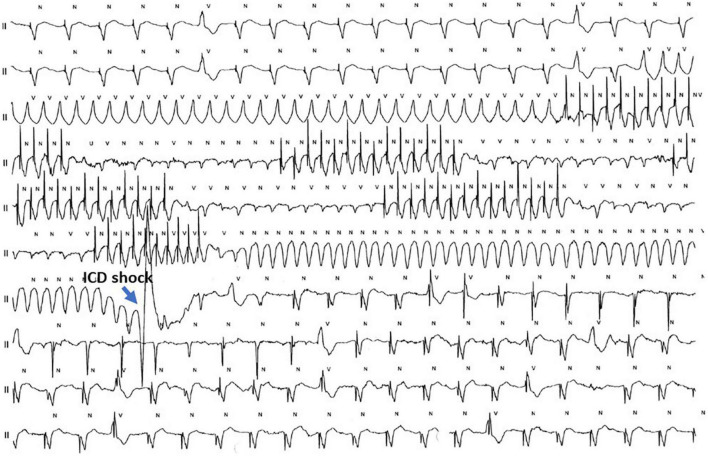
Telemetry of a 77 year-old CRT-D male patient with a dilated post-infarct heart disease, severely depressed left ventricular ejection fraction, chronic obstructive lung disease, and renal impairment who presents with electrical storm (“high-risk” profile). On the recording, the initial rhythm is a normal pacing rhythm in VDD modality with some single monomorphic premature ventricular beats (PVBs). A fast ventricular tachycardia (FVT) is triggered by the PVB. The first ATP burst of 14 pulses causes degeneration of the VT into a low-amplitude polymorphic FVT refractory to two ATP burst attempts. A 3rd ramp ATP causes degeneration into ventricular flutter which is terminated after a single ICD shock. This patient was stabilized with endovenous amiodarone and underwent transcatheter ablation on the next day.

When repeated ATPs do not terminate VT or there is immediate VT recurrence, further treatment depends on the persistence of hemodynamic stability, on VT cycle length, and the severity of underlying heart disease. If there is concern for accelerating the VT or for degeneration in VF, the best choice is to consider electrical external cardioversion, after having repeated ATP attempts with the patient under sedation. Actually, repeated ATPs may cause shortening of the CL and morphology change of the VT as well as induce VT. Further management depends on hemodynamic conditions, the characteristics of the new VT (CL and stability), and on programmed ICD settings. If the new, more rapid VT is still below the set ICD therapy window and the patient remains stable, then ATP delivery should be repeated by considering the CL of the new VT. If the accelerated VT falls within the therapy window then the ICD will intervene as programmed. Degeneration in VF will trigger ICD shock (Figure 7).

Alternatively, if the patient remains stable, further mild sedation with midazolam may be performed and beta-blocker can be administered before delivering ATPs once again (Table 1). The latter is particularly indicated in cases of incessant slow VT and mild to moderate compromised left ventricular systolic function.

#### 4.4.2 Sympathetic drive management

The autonomic nervous system plays a major role in the pathophysiology of arrhythmias potentially leading to sudden cardiac death. Sympathetic hyperactivity plays a critical role in VT onset and maintenance, thus modulation of neuro-axial efferent cardiac neurotransmission may be a target (55, 56). In case of hemodynamic instability and refractory or recurrent VT despite ATP, electrical cardioversion and antiarrhythmic drugs as well as deep sedation may be attempted to reduce the sympathetic drive. Recent experiences reported high efficacy of conversion to sinus rhythm of incessant VT in selected patients by performing percutaneous left stellate ganglion blockade (PLSGB) (57, 58).

#### 4.4.3 Left ventricle unloading

In patients with a “high risk” profile who present with sustained hemodynamically stable WCT, especially when ES, acute decompensation and cardiogenic shock may occur rapidly, especially if the first therapeutic measures are ineffective to control the arrhythmia (59). In such patients, required measures should be undertaken to consider and prepare for MCS (60). The hemodynamic support obtained from a chosen MCS is an effective rescue treatment for cardiogenic shock secondary to VT refractory to medical therapy and sedation. LV unloading by MCS in this setting improves end-organ perfusion and may contribute to sinus rhythm conversion (61, 62). MCS may provide hemodynamic support for VT ablation when a bailout transcatheter ablation procedure is needed to treat refractory VT. Moreover, MCS and catheter ablation have shown a synergic role to achieve electric and hemodynamic stabilization (63).

Various MCS techniques have been studied and used in clinical practice in secondary and tertiary centers. Intra-aortic balloon pump (IABP) is the most widely used device in low-output states. IABP decreases LV pressures and increases stroke volume, however, IABP has been recently downgraded in various guidelines (63). Percutaneous axial blood flow pump such as the Impella are increasingly used. Impella devices entrain blood from the LV and pump it into the aorta, thus unloading the LV (64). Lastly, extracorporeal membrane oxygenation extracorporeal membrane oxygenation (ECMO) is a portable modification of a cardiopulmonary bypass providing cardiopulmonary support for patients with refractory shock with or without multi-organ failure (63). Large randomized clinical trials comparing different devices and different timing strategies are lacking.

#### 4.4.4 Radiofrequency catheter ablation

Catheter ablation is an important treatment option in tertiary centers for the management of incessant VT or ES. Urgent catheter ablation is recommended in “high-risk” patients with scar-related incessant VT or ES with or without MCS support (65). Moreover, catheter ablation is recommended in patients with ischemic heart disease and recurrent effective ICD shocks due to sustained VT (66, 67). Most monomorphic VT have an origin or myocardial substrate that can be targeted for ablation. Catheter ablation risks and outcomes depend on the presence and type of structural heart disease as well as the mechanism, location, epicardial exit and acute setting of the VT (60). Catheter ablation by advanced strategies has been effectively applied to a various patient populations in the acute management of ES (65). However, catheter ablation of VT may results in various local and systemic complications, including stroke, valve damage, cardiac tamponade or AV block with a procedure-related mortality ranging from 0 to 3% (60). Moreover, urgent catheter ablation of VT is system-demanding because it requires experienced operators and advanced mapping and ablation equipment and staff support as well the need for on-call electrophysiological laboratory staff, anesthesiologists and surgical back-up. Therefore, the ablation management strategy is possible only in tertiary centers.

## 5. PHASE 4: Hospitalization and follow-up

After an ICD patient experiences sustained WCT which has been treated effectively, hospitalization ensues for completing diagnostics and implementing therapeutic measures for preventing recurrences. Further diagnostic evaluations aiming better myocardial substrate characterization include transthoracic echocardiography and/or cardiac magnetic resonance imaging. For better characterization of the arrhythmic substrate, particularly in the presence of frequent premature ventricular beats and episodes of non-sustained ventricular tachycardia, a 24–48 h 12 lead Holter electrocardiogram is useful for morphologic characterization and for the confirmation of medical therapy effectiveness (34).

Coronary artery angiography has a limited role for patients with hemodynamically stable WCT with a few exceptions. Urgent coronary angiography and, if indicated, revascularization are recommended for incessant VT or unstable patients when myocardial ischemia cannot be excluded or coronary artery disease (CAD) is suspected (34). Non-urgent coronary angiography is recommended in stable patients with or without dilated heart disease with an intermediate risk of CAD (34).

Long-term medical treatment is usually based on associating amiodarone with progressive up-titration of beta-blocker therapy (Table 4) in patients with SHD. In cases of recurrences, regardless of arrhythmia mechanism, and once reversible causes have been treated and corrected, transcatheter ablation is indicated. For recurring VT, several randomized controlled trials have demonstrated that trancatheter ablation is effective in preventing VT recurrences (68), especially if performed early on (69, 70).

**TABLE 4:** Anti-arrhythmic measures used for the prevention of WCT recurrences when the diagnosis is ventricular tachycardia.

Anti-arrhythmic drug	Dosages	Remarks and precautions
Beta-blocker therapy (metoprolol, bisoprolol, or carvedilol)*	Up-titration to maximum tolerated dose	Prevents VT and SCD in patients with SHD through modest anti-arrhythmic effect
Sotalol	Starting dose: 40-80 mg BID Target dose: 120-160 mg BID	Monitor ECG (SR, QRS duration, and QT interval) Monitor renal function Contraindicated in advanced HF disease
Amiodarone	200–400 mg daily	Monitor multi-organ side-effects As with sotalol monitor ECG parameters
Mexiletine	200–400 mg TID	2° line treatment in combination with amiodarone Caution in pts with SHD
Flecainide	50–100 mg BID	In ARVD

*Patients with SHD besides up-titration of beta-blocker therapy, optimization of heart failure medication should be performed by ensuring that the therapeutic scheme is complete (includes ARNI, an anti-aldosteronic agent, and SGLTII agent) with adequate dosages. ARVD, arrhythmogenic right ventricular dysplasia; BID, twice a day; SCD, sudden cardiac death; SHD, structural heart disease; SR, sinus rhythm; TID, three times daily; VT, ventricular tachycardia.

Planning a diagnostic and therapeutic follow-up program is of fundamental importance for the prevention and long-term management of arrhythmic recurrences. Different medical figures, including the family doctor, the general cardiologist as well as the heart failure and arrhythmia specialists, should play a concerted role in the follow-up of these patients.

## 6. Summary and conclusion

A structured and coordinated strategy is recommended for the management of every cardiovascular emergency, including hemodynamically stable WCT in ICD patients. As a first approach, basic knowledge of how to manage the ICD in this situation is important, by quickly recognizing ICD type and manufacturer, followed by application of the magnet to prevent unnecessary or inappropriate ICD shocks in a conscious patient. Differential ECG diagnosis is fundamental for further patient management as is rapid identification of potentially reversible arrhythmic triggers. Patients who present with ES, with severe underlying heart disease, heart failure symptoms, and comorbidities are “high risk” patients and should be channeled toward an accelerated management protocol that includes pre-alerting a tertiary center.

After these initial measures have been taken and the patient is stable, under mild sedation, anesthesiological and cardiological support are required for further management and appropriate anti-arrhythmic drug treatment may be delivered. Involvement of a device specialist and the ICD clinic, when available, is absolutely indicated. ICD device interrogation confirms diagnosis, and when a transvenous ICD is involved, ATP therapies may be delivered. When sustained VT is the diagnosis, specific ATP algorithms should be delivered according to VT cycle length, with particular attention in delivering ATPs that are not too aggressive to avoid conversion to VT with shorter CL or degeneration into VF, especially in the high risk patient. The proposed ATP algorithms are extrapolated from clinical studies and are empiric. These would merit evaluation through prospective multicenter studies, specifically evaluating the safety and effectiveness of delivering ATP in patients who present with lasting incessant VT (71). If the arrhythmia is refractory to the various measures performed in the ER or the ICU further management is indicated through invasive procedures, specifically urgent transcatheter ablation (less commonly urgent coronary angiography) with or without bridging with MCS.

Clinical management of ICD patients presenting with hemodynamically stable WCT depends on a coordinated multidisciplinary effort which consists in defining the best strategy on a patient-to-patient basis. Prompt recognition of the type of ICD, of arrhythmic triggers, and of patients’ general status are key for the optimal management of these challenging clinical scenarios.

## Author contributions

FR, MC, and FK contributed to the conception and design of the manuscript to its final form. AT, HB, and LS contributed to the preparation of the tables, the figures, the reference list and by reviewing for content. All authors have contributed by reviewing the manuscript critically and approving the final version.

## Appendix

### Specific responses of some ICDs when a magnetic field is detected

Specific aspects concerning application of an external magnet over an ICD can:

1. In Abbott and Boston Scientific ICDs magnet response is programmable and nominally programmed for inhibition of detection and therapies (Abbott) and only therapies in Boston Scientific transvenous ICD devices (while detection remains active). For other manufactures, magnet response is not programmable.
2. Different ICD models often release an acoustic signal when in contact with an electromagnetic interference, such as application of a magnet. The acoustic signal *does not* mean that the ICD will imminently deliver a shock.
3. Characteristics of acoustic signals are important to recognize for ICDs in which magnet response is programmable, namely for some Abbott and Boston Scientific models. In recent generation Abbott ICDs (Avant, Gallant, Entrant, and Neutrino) magnet mode initiation is indicated by emission of a 4 s tone (magnet mode termination by a 6 s higher tone). For most Boston Scientific transvenous ICDs a constant tone indicates “Monitor Only”, unless the magnet response has been programmed “Off/Ignore”. For Boston Scientific S-ICD, 60 s duration beeping confirms deactivation of detection and therapy. For older Boston Scientific ICD models, namely PRIZM, PRIZM 2, and VITALITY the acoustic signal changes from beep to continuous (therapies deactivated), while from continuous to beep signifies that the therapies are re-activated. In these older models, magnet repositioning causes switches in magnet response between “Off” and “Monitor + Therapy.”
4. In Microport (former Sorin) ICDs, pacing mode, sensor function, pacing polarity and intervals do change during magnet response. Specifically, pacing output is increased to 6V @ 1 ms for each chamber, the sensor (R-function) is disabled, if the device is in “Mode switch” pacing is performed according to permanently programmed mode independently of underlying rhythm, and, in CRT-D devices, AV delay does not change, but VV delay is set to 0 ms.
5. For some ICD devices, detection ± therapy inhibition duration do not always follow magnet application. For Microport ICDs, therapy Inhibition may extend up to 2.5 min after magnet removal if a charge occurred just before magnet application. For Biotronik ICDs, after 8 h of continuous magnet application, tachy-detection/therapies are automatically re-enabled.
